# Multi-Scale Characterization of Interfacial Adhesion and Material Selection for Crack Sealants in High-Altitude Airport Asphalt Pavements

**DOI:** 10.3390/ma19153329

**Published:** 2026-08-05

**Authors:** Shuqi Li, Yukun Zhou, Xiaoyi Du, Bing Hui

**Affiliations:** 1 School of Highway, Chang’an University, Xi’an 710064, China; shuqili@chd.edu.cn (S.L.); yukun@chd.edu.cn (Y.Z.); huibing@chd.edu.cn (B.H.); 2 The Key Laboratory of Intelligent Construction and Maintenance of CAAC, Chang’an University, Xi’an 710064, China

**Keywords:** crack sealant, asphalt pavement, interfacial adhesion failure, molecular dynamics simulation, multi-scale analysis

## Abstract

**Highlights:**

- All adhesion and mechanical indicators rose monotonically with modifier content; sealant C ranked optimal by CRITIC-TOPSIS.
- Electrostatic forces drive sealant–aggregate adhesion; van der Waals forces govern sealant–asphalt adhesion.
- A multi-scale chain linking phase morphology, intermolecular forces, interface energy and macroscale mechanics was established.

**Abstract:**

Asphalt pavements at high-altitude airports endure prolonged extreme low temperatures and large diurnal swings, imposing stringent demands on crack sealants, whose multi-scale adhesion failure mechanism remains unclear. Three SBS and crumb-rubber-composite-modified sealants, designated A, B and C, were characterized through surface free energy tests, pull-off and shear tests, fluorescence microscopy, FTIR and molecular dynamics simulations. Cross-scale correlation analysis and CRITIC-TOPSIS were applied to link and rank the sealants across scales. Work of cohesion, work of adhesion, pull-off strength and shear strength all rose monotonically with modifier content, and sealant C exhibited a 38.5% higher work of cohesion and a 52.4% lower CV_φ_ than sealant A. Molecular dynamics simulations showed that electrostatic forces drove sealant–aggregate adhesion while van der Waals forces governed sealant–asphalt adhesion, with a simulation–experiment deviation of only 2.88–5.74%. A level-by-level transmission linked phase-morphology uniformity, intermolecular interaction, interfacial energy and macroscopic mechanical performance. Sealant C achieved a CRITIC-TOPSIS index of 1.000, far above 0.271 for B and 0.000 for A, and is recommended as the preferred material for crack sealing of high-altitude airport asphalt pavements.

## 1. Introduction

Airport asphalt pavements are subjected throughout their service life to the combined effects of aircraft loading, thermal cycling, and ultraviolet radiation, and cracking is the most prevalent and destructive form of distress [1,2]. Environmental conditions in high-altitude regions are particularly severe: extreme low temperatures and large diurnal temperature variations accelerate crack initiation and propagation [3,4], and crack development exhibits a clear temperature-threshold effect [5,6]. Crack sealing has become the primary maintenance strategy for asphalt pavement cracks owing to its ease of application and cost-effectiveness. Min et al. conducted a systematic review of crack sealing technologies and identified interfacial debonding and shear failure as the principal failure modes [7]. SBS and crumb-rubber-composite-modified asphalt crack sealants are widely used because they combine low-temperature ductility with high-temperature stability [8,9]. However, adhesion failure during service involves a hierarchical process spanning microstructural morphology, molecular interactions, and macroscopic mechanical response [10,11], and the underlying mechanisms remain poorly understood.

Extensive research has been conducted on the interfacial adhesion performance of crack sealants at both macroscopic and microscopic scales. Regarding macroscopic performance evaluation, conventional empirical indicators fail to capture the thermodynamic nature of interfacial adhesion [12]. Chen et al. established a cross-scale correlation between molecular debonding behavior and pull-off strength using surface free energy theory [13], and Wang et al. calculated the interfacial work of adhesion of crack sealants in seasonally frozen regions from contact angle measurements [14]. Yang et al. systematically investigated the effects of temperature and crack width on the shear strength of sealant–aggregate interfaces [15], while He et al. reported that thermal cycling significantly affects the interfacial stability of crack sealants [10]. Yang et al. examined the progressive effects of UV aging on hot-poured crack sealant performance [16], and Ma et al. and Guo et al. evaluated the mechanical properties of epoxy-based and hot-poured crack sealants, respectively [17,18]. At the microscale, Zhang et al. used fluorescence microscopy to investigate the dispersion morphology and modification effect of SBS in the asphalt matrix [19], and Yan et al. and Zhang et al. characterized the blending state of modifiers and chemical structural changes using FTIR [20,21]. However, these studies were each confined to a single observation scale; the causal relationship between thermodynamic parameters and mechanical strength has not been quantitatively established, and the link between microstructural observations and macroscopic performance differences has largely remained at the level of qualitative inference without a complete causal chain.

To overcome the limitations of experimental characterization at the atomic scale, molecular dynamics (MD) simulation has been introduced as a quantitative analytical tool for studying the interfacial behavior of asphalt-based materials. Zhang et al. and Du et al. investigated strategies for constructing modified asphalt molecular models and evaluating compatibility, respectively [22,23]. Yao et al. calculated fundamental physical properties of asphalt by MD simulation and validated the model [24], while Li et al. combined molecular simulation with microscale experiments to study the interfacial adhesion thermodynamics of modified asphalt [25]. Regarding interfacial adhesion mechanisms, Huang et al. revealed the relationship between molecular diffusion capacity and adhesion strength at the asphalt–aggregate interface [26]; Cheng et al. calculated the work of adhesion at asphalt–mineral interfaces and analyzed temperature effects [27]; Ma et al. examined the influence of simulation details on the accuracy of interface energy calculations [28]; and Gao et al. investigated the effect of crumb rubber content on the interfacial adhesion characteristics of modified asphalt [29]. Xing et al. and Yao et al. each conducted systematic reviews of MD simulation research on asphalt pavement materials [30,31]. Nevertheless, existing MD studies have focused primarily on asphalt binders or asphalt mixture systems; applications to the dual-interface system comprising crack sealant–aggregate and crack sealant–asphalt interfaces remain limited, and the molecular driving mechanisms of crack sealant interfacial adhesion failure have not been elucidated.

Given the limitations of single-scale studies, multi-scale integration has emerged as an important approach for elucidating material interfacial behavior. Chen et al. analyzed pavement moisture damage resistance across three scales—asphalt binder, asphalt–aggregate, and asphalt mixture [32]; Gong et al. combined MD simulation with macroscopic experiments to study asphalt self-healing [33]; and Wu et al. and Wang et al. examined warm-mix asphalt moisture damage and asphalt–aggregate interfacial behavior, respectively, from multi-scale perspectives [34,35]. The CRITIC-TOPSIS method offers an objective advantage in the comprehensive evaluation of pavement materials, as its weights are entirely data-driven [36]. However, no study has yet systematically integrated microstructural characterization, molecular simulation, and macroscopic testing for crack sealants; quantitative correlations across scales have not been established; and the hierarchical transmission mechanism of interfacial adhesion failure remains unclear.

Building on this analysis, the present study selected three low-temperature composite-modified asphalt crack sealants with varying SBS and crumb rubber contents. Surface free energy measurements, pull-off tests, and shear tests were conducted to quantify interfacial adhesion and mechanical performance. Fluorescence microscopy and FTIR spectroscopy were used to characterize microstructural phase morphology. MD simulations were performed to calculate interaction energies and the work of adhesion at both the sealant–aggregate and sealant–asphalt interfaces. Spearman correlation analysis was then applied to examine the consistency of indicators across scales and to elucidate the multi-scale mechanisms of interfacial adhesion failure. Finally, CRITIC-TOPSIS was employed for objective comprehensive evaluation, providing a scientific basis for the selection of crack sealing materials for high-altitude airport asphalt pavements.

## 2. Materials and Methods

### 2.1. Materials

Three low-temperature composite-modified asphalt crack sealants, designated sealant A, sealant B, and sealant C, were selected based on the service environment characteristics of high-altitude airports. All three materials were prepared by co-blending SBS modifier and crumb rubber in a continuous phase of matrix asphalt; the mass fractions of the main components are listed in Table 1. The SBS and crumb rubber contents increase in a gradient from A to C, with a corresponding reduction in asphalt content, thereby forming a comparative series with systematically varying cross-linking network densities. Basic performance tests were conducted on all three sealants in accordance with JT/T 740-2015 [37]; the results are presented in Table 2, and all measured properties meet the technical requirements for low-temperature crack sealants.

### 2.2. Contact Angle and Surface Free Energy Test Methods

**Figure 1:**
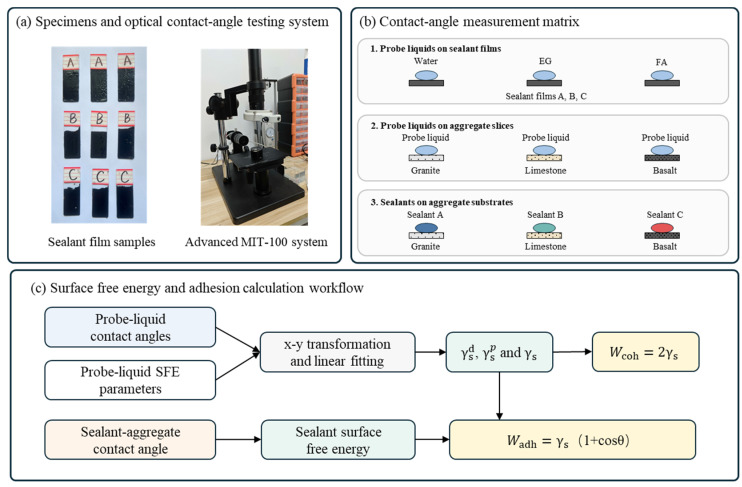
Contact angle measurement setup.

To quantitatively evaluate the cohesive strength of the crack sealants and their interfacial adhesion to aggregates from a thermodynamic perspective, surface free energies of both the sealants and the aggregate specimens were determined by the sessile-drop method using the Owens–Wendt model. All measurements were performed at 25 °C using an Advanced MIT-100 optical contact angle measurement system (Shanghai Chugong Industrial Co., Ltd., Shanghai, China); the contact angle geometry, sealant film specimens, and test setup are shown in Figure 1. Sealant specimens were prepared as follows: glass slides were dried at 120 °C for 1 h, immersed in the molten sealant for 60 s to form a uniform coating, leveled, held at 120 °C for 3 min, and then cured at 25 °C for 24 h to relieve residual stress. Distilled water, ethylene glycol, and formamide were used as probe liquids; four parallel specimens were prepared for each sealant-liquid combination and the results were averaged. Basalt, granite, and limestone aggregates were selected; specimens were cut, polished, and ultrasonically cleaned with deionized water, then subjected to contact angle measurements using the same procedure. For the sealant-on-aggregate contact angle, each sealant was heated above its softening point to a fluid state and deposited onto the polished aggregate surface using a microsyringe; after the droplet spread and stabilized, the contact angle was measured at 25 °C. This hot sessile-drop technique is widely used for characterizing the wettability of bituminous materials on mineral substrates [38]. Four replicate measurements were made for each sealant–aggregate combination. The work of cohesion of each sealant was calculated from its total surface free energy, and the work of adhesion between each sealant and aggregate was derived from the sealant surface free energy and the corresponding sealant–aggregate contact angle; these quantities serve as thermodynamic indicators of cohesive capacity and interfacial bonding ability, respectively.

The surface energy parameters of the probe liquids are listed in Table 3. When the solid–liquid–gas three-phase system reaches interfacial energy equilibrium, the interfacial tensions of all phases satisfy the Young equation, Equation (1). The interfacial tension comprises a dispersive component and a polar component, Equation (2). Combining and rearranging these two equations into linear form, Equation (3), the dispersive and polar components of each material were determined by linear regression of the contact angle data. Because the partition of the total surface free energy into dispersive and polar components depends on the selected probe-liquid combination, the subsequent interpretation is based primarily on the total surface free energy and the derived works of cohesion and adhesion [39]. On this basis, the work of cohesion of the sealant was calculated using Equation (4) and the work of adhesion between the sealant and aggregate using Equation (5).

(1) $$\gamma_{s-l}-\gamma_{s-g}+\gamma_{l-g}\cos\theta =0$$

where $\gamma_{s-l}$ is the solid–liquid interfacial tension, mJ/m^2^; $\gamma_{s-g}$ is the solid–gas interfacial tension, mJ/m^2^; $\gamma_{l-g}$ is the liquid–gas interfacial tension, mJ/m^2^; and *θ* is the contact angle.

(2) $$\gamma_{s-l}=\gamma_{l-g}+\gamma_{s}^{0}-2\sqrt{\gamma_{s}^{d}\gamma_{l-g}^{d}}+2\sqrt{\gamma_{s}^{p}\gamma_{l-g}^{p}}$$

where $\gamma_{s}^{0}$ is the surface free energy of the solid in vacuum, mJ/m^2^, superscripts *d* and *p* denote the dispersive and polar components, respectively.

(3) $$\frac{\left(1+\cos\theta \right)}{2}\cdot \frac{\gamma_{l}}{\sqrt{\gamma_{l}^{d}}}=\sqrt{\gamma_{s}^{p}}\cdot \sqrt{\frac{\gamma_{l}^{p}}{\gamma_{l}^{d}}}+\sqrt{\gamma_{s}^{d}}$$

(4) $$W_{coh}=2\gamma_{s}$$

where $W_{coh}$ is the work of cohesion of the sealant, mJ/m^2^.

(5) $$W_{adh}=\gamma s(1+\cos\theta )$$

where $W_{adh}$ is the work of adhesion between sealant and aggregate, mJ/m^2^, and *θ* is the contact angle between sealant and aggregate.

#### 2.2.1. Macroscopic Mechanical Tests

##### Pull-Off Strength Test

**Figure 2:**
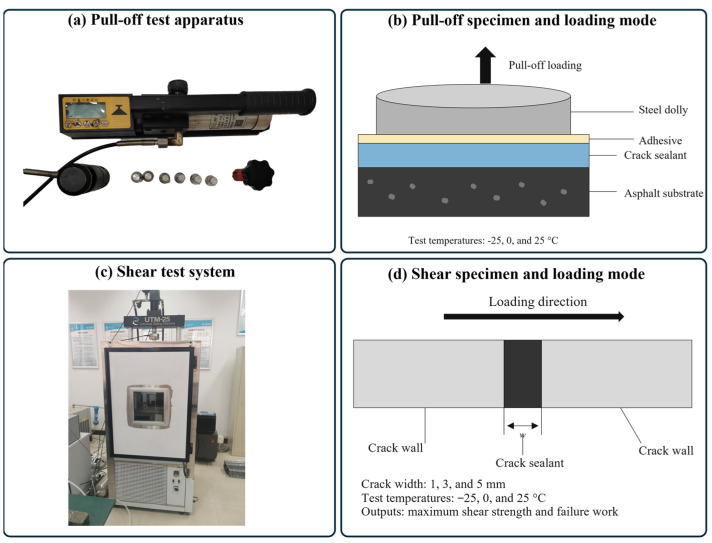
Macroscale mechanical test setup: (**a**) pull-off test apparatus; (**b**) pull-off specimen and loading mode; (**c**) shear test system; (**d**) shear specimen and loading mode.

To evaluate the bond strength of the sealant–aggregate interface under normal tensile loading, pull-off strength tests were conducted in accordance with ASTM D4541-09 [40] using an XH-M portable adhesion tester (Beijing Tiandi Spark Technology Development Co., Ltd., Beijing, China), as shown in Figure 2. AC-13 Marshall specimens were used as substrates. Each sealant was heated to its application temperature, uniformly applied to the specimen surface, and allowed to cool and set naturally. A 20 mm diameter dolly was coated with AB adhesive and bonded to the coated surface; excess adhesive at the perimeter was removed, and the assembly was conditioned at the target temperature for at least 2 h. A progressive vertical tensile load was then applied until the dolly detached from the specimen surface; the peak force and failure mode were recorded. Test temperatures of −25 °C, 0 °C, and 25 °C were selected to reflect the actual temperature range of high-altitude airport pavements. Three parallel specimens were tested per condition and the results were averaged.

##### Shear Performance Test

Airport pavements are subjected to significant interfacial shear forces during aircraft take-off and landing, and insufficient shear resistance between the crack sealant and the crack walls can lead to interfacial delamination failure. Interfacial shear tests were conducted in accordance with ASTM C882/C882M-20 [41]. Specimens were cut from AC-13 asphalt concrete slabs to dimensions of 50 mm × 100 mm × 50 mm; the cut surface was designated to simulate the crack interface, and all specimens were conditioned at constant temperature for at least 5 h to achieve interfacial stability. Testing was performed using a UTM-25 universal testing machine (IPC Global, Melbourne, Australia) (Figure 2); load was applied at a constant rate of 50 mm/min through a dedicated fixture until failure. Two sets of tests were conducted with crack width and test temperature as the variables: crack widths of 1, 3, and 5 mm, and temperatures of −25 °C, 0 °C, and 25 °C. Three parallel specimens were tested per condition and the results were averaged. Shear strength was calculated using Equation (6). Failure work was determined by integrating the shear force–displacement curve to quantify the energy required for interfacial failure under each test condition.

(6) $$\tau =\frac{F\cdot \sin\alpha }{S}$$

where τ is the shear strength, MPa; F is the applied vertical force, N; S is the shear plane area, mm^2^, and α is the oblique shear angle, 45° in this study.

#### 2.2.2. Microstructural Characterization

##### Fluorescence Microscopy

**Figure 3:**
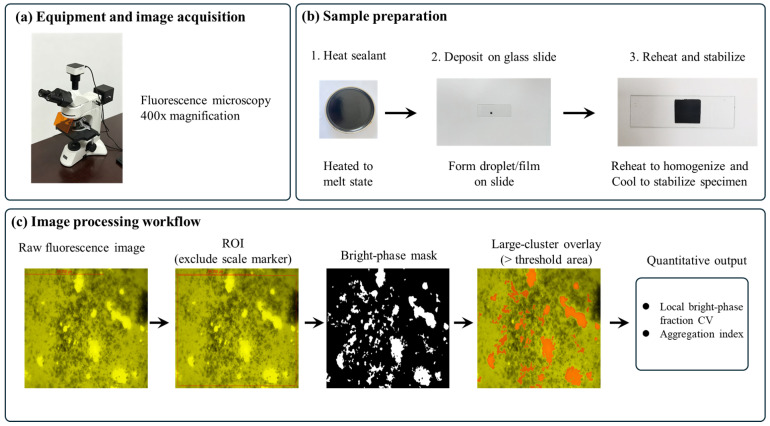
Fluorescence microscopy specimen preparation and image-analysis workflow.

The macroscopic mechanical performance of crack sealants is closely related to their microstructural phase morphology. The microstructural morphology of all three sealants was examined using an LW300LFT fluorescence microscope (Xi’an Cewei Optoelectronic Technology Co., Ltd., Xi’an, China); four specimens per sealant were prepared and fluorescence images were acquired at 400× magnification. Specimen preparation followed the same procedure as described in Figure 3: each sealant was heated to its fluid state and applied to a glass slide to form a uniform thin film.

To quantitatively characterize the spatial distribution uniformity of the fluorescent phase, all acquired images were analyzed using the digital image analysis workflow illustrated in Figure 3. Regions containing the red scale bar and a 2% edge margin were first excluded; the remaining area was designated as the region of interest (ROI). Within the ROI, the bright phase was extracted by thresholding the top 15% brightest pixels in the CLAHE-enhanced (clip limit 2.0, tile 8 × 8) HSV value channel, with a minimum saturation of 35; objects smaller than 0.002% of the ROI area were removed as noise. The ROI was divided into 8 × 8 = 64 equal subregions, and the local bright-phase fraction of each subregion was calculated using Equation (7).

(7) $$\varphi_{i}=\frac{A_{b,i}}{A_{i}}$$

where $\varphi_{i}$ is the local bright-phase fraction in the i-th subregion, $A_{b,i}$ is the bright-phase area in the i-th subregion, and $A_{i}$ is the total area of the i-th subregion.

The coefficient of variation of the local bright-phase fraction, CV_φ_, quantifies the degree of spatial non-uniformity of the fluorescent phase, Equation (8); a higher CV_φ_ indicates greater heterogeneity in the spatial distribution of the fluorescent phase.

(8) $$CV_{\varphi }=\frac{\sigma_{\varphi }}{\mu_{\varphi }}$$

where $\mu_{\varphi }$ and $\sigma_{\varphi }$ are the mean and standard deviation of the local bright-phase fraction among all subregions.

The aggregation index AI characterizes the extent to which the fluorescent phase concentrates into large-scale clusters, Equation (9); a higher AI indicates a stronger tendency of the fluorescent phase to form large-scale agglomerates.

(9) $$AI=\frac{A_{large}}{A_{bright}}\times 100\%$$

where $A_{large}$ is the total area of large bright clusters (connected domains ≥ 0.05% of the ROI area), and $A_{bright}$ is the total bright-phase area in the ROI.

##### Fourier-Transform Infrared Spectroscopy

To verify the chemical compositional consistency of the three crack sealants and the blending state of the SBS modifier, chemical structural analysis was performed by Fourier-transform infrared spectroscopy (FTIR) using a Shimadzu IRTracer-100 spectrometer (Shimadzu Corporation, Kyoto, Japan) equipped with a MIR TGS detector. Spectra were collected over a wavenumber range of 500–4000 cm^−1^. Functional groups were identified from the positions and intensities of characteristic absorption bands to assess whether the SBS modifier had been successfully incorporated into the asphalt matrix; measurements were conducted on all three sealant specimens.

#### 2.2.3. Molecular Dynamics Simulation

Macroscopic tests alone are insufficient to reveal the microscopic mechanisms of interfacial adhesion failure in crack sealants. Molecular models of the crack sealants, aggregates, and asphalt were therefore constructed in Materials Studio 2019, and MD simulations were performed to quantify interfacial adhesion and cohesive properties at the atomic scale, providing a molecular-level interpretation of the macroscopic experimental results. The COMPASS II force field was applied throughout, with three-dimensional periodic boundary conditions imposed on all models.

**Figure 4:**
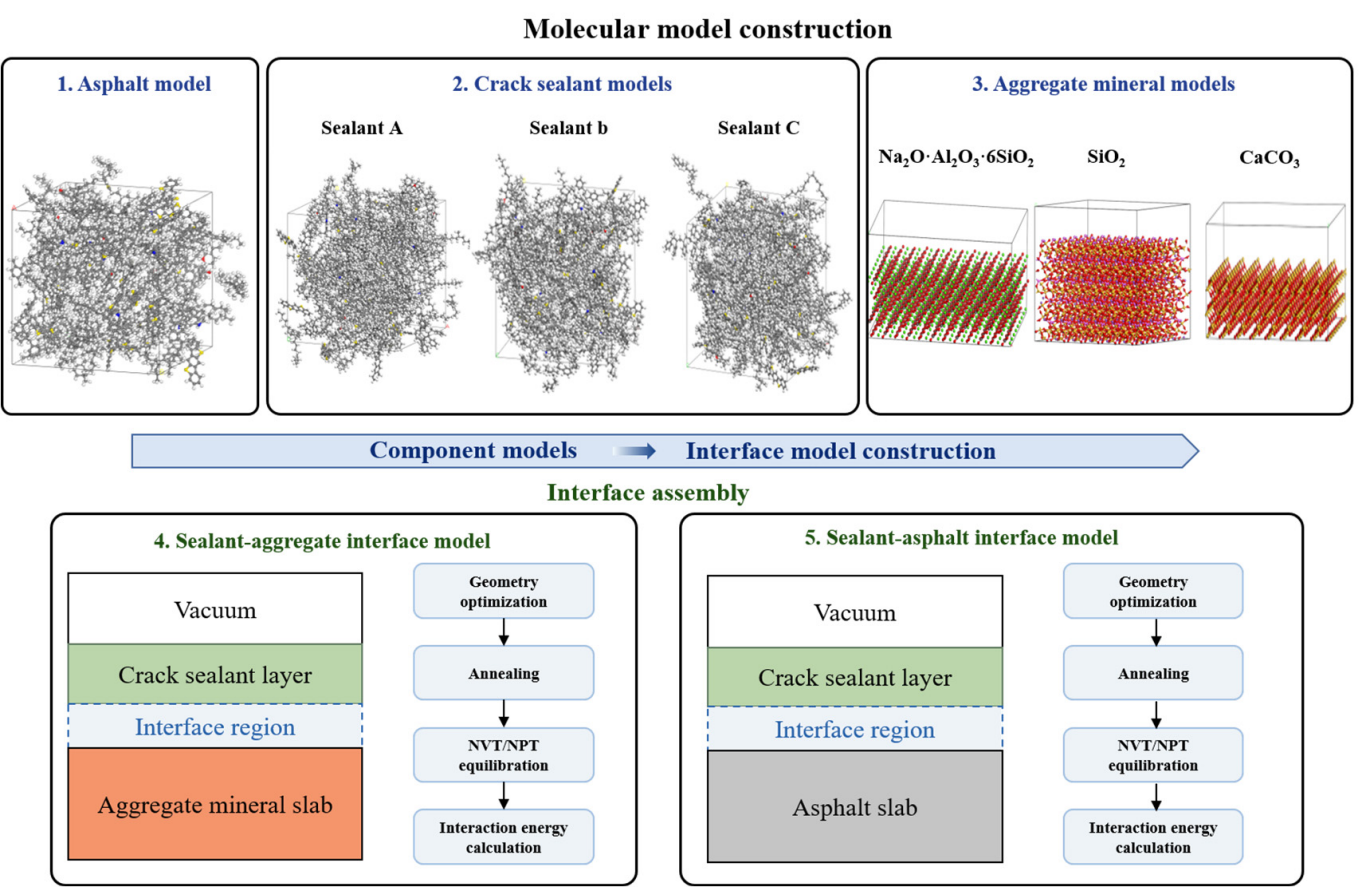
Molecular model optimization schematic.

The optimization workflow is illustrated in Figure 4. Each model was first geometry-optimized (Smart algorithm, 10,000 iterations, 12.5 Å cutoff, atom-based van der Waals summation, Ewald electrostatics), then relaxed under the NVT ensemble for 200 ps (298 K, Berendsen thermostat, 1 fs time step), and subsequently equilibrated under the NPT ensemble for 100 ps (Andersen thermostat at 298 K, Berendsen barostat at 0.1 MPa). Equilibrium was confirmed by the simultaneous convergence of potential energy, simulation-box dimensions, and density, with the temperature stabilized at 298 K (fluctuation < 5 K).

##### Molecular Model Construction and Optimization

Asphalt is a multi-component, multi-phase complex system. Based on the SARA four-component classification defined in ASTM D4124-09 [42], the asphalt molecular system was constructed using the 4-component, 12-molecule model proposed by Li et al. [43], comprising 8, 24, 32, and 8 molecules of saturates, aromatics, resins, and asphaltenes (a molecule-count ratio of 8:24:32:8, corresponding to mass fractions of 11.1%, 31.9%, 39.7%, and 17.3%); model parameters are listed in Table 4. The assembled cell contained 72 molecules with an overall composition of C_2325_H_3192_N_10_O_8_S_37_ and a total mass of 32,544 g/mol. An amorphous cubic unit cell with three-dimensional periodic boundary conditions was built using the Amorphous Cell module at an initial density of 0.6 g/cm^3^ and 298 K. Model optimization proceeded through three sequential stages: geometry optimization, NVT ensemble equilibration, and NPT ensemble relaxation. Geometry optimization was performed in the Forcite module for 10,000 iterations with a cutoff radius of 12.5 Å, using the Smart algorithm, the Ewald summation for electrostatic interactions, and the atom-based method for van der Waals interactions. The NVT stage was conducted at 298 K with a 1 fs time step for a total of 200 ps using the Berendsen thermostat. The NPT stage employed an Andersen thermostat at 298 K and a Berendsen barostat at 0.1 MPa, with a relaxation time of 0.5 ps, a 1 fs time step, and a total duration of 100 ps; density convergence was used as the equilibration criterion. After optimization, the asphalt unit cell converged to dimensions of 37.98 Å × 37.98 Å × 37.98 Å.

The sealant molecular models were constructed by adding SBS and crumb rubber molecules at the mass fractions specified in Table 1 to the asphalt model. SBS was represented as a styrene–butadiene–styrene triblock copolymer with polymerization degrees of n = 6 and m = 3. Both modifiers were modeled as copolymer chains. Styrene–butadiene rubber (SBR), which constitutes 60–75% of the crumb rubber and serves as its primary modifying component, was selected as the representative. In line with molecular-simulation studies of rubber-modified asphalt [44], SBR was represented as a random styrene–butadiene copolymer chain built from 4 styrene (C_8_H_8_) and 12 butadiene (C_4_H_6_) repeat units, giving a chain composition of C_80_H_104_, a chain mass of 1065.7 g/mol, and mass fractions of 39.1% styrene and 60.9% butadiene, as listed in Table 5. The three sealant models were assembled in the Amorphous Cell module at the prescribed mass fractions and optimized following the same procedure as the asphalt model.

Basalt, granite, and limestone are polymineralic aggregates whose interfacial behavior is governed largely by the surface chemistry of their principal mineral phases. Their surfaces were modeled by representative end-member minerals, albite (Na_2_O·Al_2_O_3_·6SiO_2_), quartz (SiO_2_), and calcite (CaCO_3_), which correspond to alkaline aluminosilicate, acidic silica, and carbonate surface chemistries. Single-phase mineral surrogates of this kind have been used in molecular simulations of asphalt–aggregate interfaces [45]. The corresponding crystal unit cells were loaded from the model library; lattice parameters are listed in Table 6. Each unit cell was cleaved along its most stable surface: the (001) plane for SiO_2_, the (104) plane for CaCO_3_, and the (001) plane for Na_2_O·Al_2_O_3_·6SiO_2_; lattice angles were uniformly set to α = β = γ = 90°. The in-plane repeat distances of the cleaved surfaces were 7.172 Å for Na_2_O·Al_2_O_3_·6SiO_2_, 4.915 Å for SiO_2_, and 5.125 Å for CaCO_3_; the supercell dimensions in Table 7 were obtained from these in-plane repeats and the corresponding multipliers. Since the surface area of a single cleaved unit cell is considerably smaller than that of the sealant model, supercell expansion was performed to match the two surface areas; supercell parameters are listed in Table 7. A 10 Å vacuum layer was added above each surface to eliminate non-physical periodic interactions along the z-axis. All aggregate models were stabilized through 10,000 steps of geometry optimization followed by NVT/NPT relaxation.

To validate the constructed molecular models, four thermodynamic parameters were used for comprehensive assessment: density, cohesive energy density, solubility parameter, and radial distribution function. Density, as a fundamental physical property, was compared with experimentally measured values to assess whether the model configuration had reached a realistic density level. Cohesive energy density is defined as the energy per unit volume required to completely dissociate a condensed-phase substance into isolated gas-phase molecules and reflects the overall strength of intermolecular interactions. The solubility parameter is defined as the square root of the cohesive energy density and characterizes thermodynamic compatibility between substances, Equation (10). Because intermolecular interactions in asphalt and crack sealant systems arise from both van der Waals and electrostatic forces, the solubility parameter can be further decomposed into van der Waals and electrostatic components. The radial distribution function g(r) is defined as the ratio of the probability density of finding a particle at distance r from a reference particle to that in a fully disordered system, Equation (11), and is used to assess the degree of structural order and overall model reliability.

(10) $$\delta =\sqrt{CED}=\sqrt{\frac{E_{coh}}{V}}$$

where δ is the solubility parameter (J/cm^3^)^0.5^; CED is the cohesive energy density, J/m^3^; $E_{coh}$ is the cohesive energy, kcal/mol; and V is the model volume, Å^3^.

(11) $$g(r)=\frac{1}{\rho N}<\sum_{i\neq j}\delta (r-r_{ij})>$$

where g(r) is the radial distribution function, ρ is the system density, kg/m^3^, N is the number of particles, and r is the radial distance, m.

##### Interface Model Construction and Performance Parameter Calculation

Upon sealing a pavement crack, two potential adhesion interfaces are formed: the sealant–aggregate interface and the sealant–asphalt interface. Bilayer interface models were constructed using the Build Layers module. For the sealant–aggregate interface model, the aggregate was placed as the first layer and the sealant as the second, with interfacial dimensions defined by the aggregate model. A 50 Å vacuum layer was added above the sealant to prevent non-physical interactions between the aggregate bottom and the sealant top arising from z-direction periodic boundary conditions. The sealant–asphalt interface model was constructed by the same method, with asphalt as the first layer and the sealant as the second. All interface models were subjected to combined geometry optimization and NVT/NPT ensemble relaxation to eliminate high-energy configurations; performance parameters were calculated after the system energy had converged to its minimum state.

The mean square displacement (MSD) of the whole crack-sealant molecular system characterizes the dynamic migration of molecules in the interfacial region; the diffusion coefficient (DC) was obtained by linear fitting of the MSD curve over the 0–60 ps interval using Equation (12). A smaller MSD reflects more restricted molecular chain segment mobility, which is consistent with a stronger constraint imposed by the interface.

(12) $$MSD(t)=<|r(t)-r(0){|}^{2}>,\;\;DC=\frac{1}{6}\underset{t\to \infty }{\lim}\frac{d}{dt}MSD(t)$$

where r(t) is the particle position at time t, ps; r(0) is the initial position, and DC is the diffusion coefficient, Å^2^/ps.

Based on the atomic model, the surface free energy of the crack sealant was calculated from the potential energy difference between the full-periodic and confined models divided by the newly formed surface area; the work of cohesion is equivalent to twice the surface free energy, Equation (13).

This potential-energy formulation, in which the entropic contribution is not treated explicitly, is well established for molecular-dynamics surface-energy calculations of polymeric materials [46,47]; the energies used were time-averaged over the equilibrated production stage.

(13) $$\gamma_{s}=\frac{E_{confined}-E_{bulk}}{2A},\;\;W_{coh}=2\gamma_{s}$$

where $\gamma_{s}$ is the surface free energy, mJ/m^2^; $E_{confined}$ and $E_{bulk}$ are the potential energies of the confined and full-periodic sealant models, kcal/mol; and A is the newly formed surface area, Å^2^.

The interfacial interaction strength of the sealant–aggregate interface was characterized by the interface energy, defined as the difference between the total potential energy of the bilayer system and the sum of the potential energies of the individual isolated layers; the work of adhesion was then derived from the interface energy and the interfacial cross-sectional area using Equations (14) and (15).

(14) $$E_{int}=E_{total}-E_{sealant}-E_{aggregate}$$

(15) $$W_{adh}=-\frac{E_{int}}{A}\times 694.8$$

where $E_{int}$ is the interaction energy, kcal/mol; $E_{total}$ is the total potential energy of the interface system, kcal/mol; $E_{sealant}$ and $E_{aggregate}$ are the potential energies of isolated sealant and aggregate layers, kcal/mol; A is the cross-sectional area of the interface, Å^2^; $W_{adh}$ is the work of adhesion, mJ/m^2^; and 694.8 is the unit conversion factor.

Interface energy calculations were performed by MD simulation under the NVT ensemble for 100 ps at simulation temperatures of 253 K, 273 K, and 293 K; the potential energy of each layer was extracted after the system reached a steady state. For all systems, the reported structural, energetic, and interfacial properties were obtained from one fully equilibrated trajectory and determined by time-averaging over the production stage rather than from instantaneous configurations, after the convergence of potential energy, box dimensions, and density had been confirmed.

#### 2.2.4. Multi-Scale Correlation Analysis Method

Interfacial adhesion failure of crack sealants in pavement cracks involves a hierarchical process spanning from molecular-scale interfacial interactions to macroscale mechanical failure; single-scale experiments or simulations alone are insufficient to fully elucidate the underlying mechanism. To establish linkages among indicators across scales, correlation analysis was applied to two pairs of datasets. The first pair comprised the corresponding indicators from surface free energy measurements and MD simulations, and the second pair comprised the molecular-scale energy parameters and the macroscopic mechanical performance indicators. For each pair, the Spearman rank correlation coefficient ρ, reflecting agreement in ordering, and the Pearson correlation coefficient r, reflecting linear closeness, were computed. As only three formulations were compared (n = 3), the coefficients are used descriptively to characterize the consistency between scales, and no statistical inference such as *p*-values or confidence intervals is drawn. Building on these results, the fluorescence microscopy findings on modifier dispersion morphology and the MD data on intermolecular interaction mechanisms were integrated to elucidate the hierarchical transmission pathway by which microstructural phase morphology governs macroscopic mechanical response through intermolecular forces and interfacial energy parameters, thereby revealing the multi-scale mechanisms of crack sealant interfacial adhesion failure.

#### 2.2.5. CRITIC-TOPSIS Comprehensive Evaluation Method

To objectively evaluate the three crack sealants in a comprehensive manner, the CRITIC objective weighting method was combined with TOPSIS ideal-solution ranking. Unlike conventional approaches that rely on expert-assigned weights, the CRITIC method extracts weights directly from the variability and conflict among indicator data, thereby eliminating subjective intervention. Evaluation indicators were selected according to the following principles: qualitative characterization indicators were excluded; intermediate parameters used solely for model validation that do not directly reflect service performance were excluded; and where experimental and simulation results were highly consistent for equivalent indicators, only experimental values were retained to avoid information redundancy. Based on these criteria, four indicators were selected as evaluation criteria: work of cohesion, work of adhesion, pull-off strength, and shear strength, representing the material’s cohesive resistance, interfacial bonding capacity, and macroscopic load-bearing performance, respectively.

Raw data were subjected to min–max normalization, after which the CRITIC method was applied to calculate the information content of each indicator and determine the objective weights, Equation (16). The positive and negative ideal solutions were then identified from the weighted normalized matrix $V_{ij}=w_{j}\cdot z_{ij}$, and the TOPSIS comprehensive evaluation index was calculated using Equation (17).

(16) $$C_{j}=\sigma_{j}\underset{k=1}{\sum^{n}}(1-r_{jk}),\;\;w_{j}=\frac{C_{j}}{\sum_{j=1}^{n}C_{j}}$$

where $\sigma_{j}$ is the standard deviation of indicator j, $r_{jk}$ is the Pearson correlation coefficient between indicators j and k, and $w_{j}$ is the objective weight.

(17) $$C_{i}^{*}=\frac{D_{i}^{-}}{D_{i}^{+}+D_{i}^{-}},\;\;D_{i}^{\pm }=\sqrt{\underset{j=1}{\sum^{n}}{\left(V_{ij}-V_{j}^{\pm }\right)}^{2}}$$

where $D_{i}^{+}$ and $D_{i}^{-}$ are the Euclidean distances from sealant i to the positive and negative ideal solutions, and $C_{i}^{*}\in [0,1]$, with higher values indicating better comprehensive performance.

## 3. Results and Discussion

### 3.1. Macroscopic Adhesion and Mechanical Performance of Crack Sealants

#### 3.1.1. Contact Angle and Surface Free Energy

**Figure 5:**
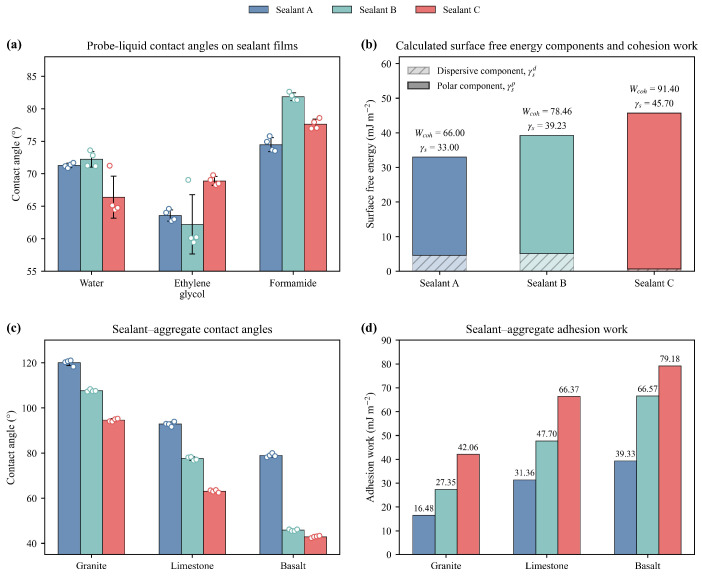
Contact angle and surface energy analysis of crack sealants: (**a**) probe-liquid contact angles on sealant films; (**b**) surface free energy components and cohesion work; (**c**) sealant–aggregate contact angles; (**d**) sealant–aggregate adhesion work.

The average contact angles of the three crack sealants with the probe liquids are shown in Figure 5a; the derived surface free energy components and work of cohesion are presented in Figure 5b, and the full set of thermodynamic parameters is listed in Table 8. The total surface free energy of the three sealants increased in the order A < B < C, reflecting the progressively stronger intermolecular interactions introduced by higher modifier content. The work of cohesion of sealant C was 38.5% and 16.5% higher than that of sealants A and B, respectively; this increased systematically with modifier content, consistent with the denser physical cross-linking network formed at higher SBS and crumb rubber contents. The surface free energies of the aggregates decrease in the order limestone > granite > basalt, consistent with their respective mineral polarity and surface pore characteristics. Although basalt, the aggregate most commonly used in high-altitude airport pavements, exhibits the lowest surface free energy, its alkaline surface active sites yield the smallest contact angle and best wettability with the sealants, which favors the establishment of stable interfacial adhesion under actual service conditions.

Contact angle images of the sealant–aggregate combinations are shown in Figure 5c. For a given sealant, the contact angle with basalt is smallest and that with granite is largest; for a given aggregate, sealant C consistently exhibits the lowest contact angle. The contact angle of the sealant C–basalt system is as low as 42.90°, whereas that of the sealant A–granite system reaches 120.04°, a difference of 77.14°. According to wetting theory, a contact angle below 90° allows the liquid phase to spontaneously penetrate into the microscopic pores of the solid surface and form mechanical interlocking; both the sealant C–basalt and sealant C–limestone systems satisfy this condition, which is favorable for forming stable interfacial structures in high-altitude airport pavements.

#### 3.1.2. Work of Cohesion and Work of Adhesion

The works of cohesion of sealants A, B, and C were 66.00, 78.46, and 91.40 mJ/m^2^, respectively, as listed in Table 8. The work of cohesion represents the critical energy required to overcome intermolecular forces when separating a bulk material and directly reflects the cohesive failure threshold. Sealant C exceeded sealant A by 25.40 mJ/m^2^ at an increase of 38.5%, consistent with the dense three-dimensional physical cross-linking network formed at its higher SBS and crumb rubber contents. The increased density of molecular chain entanglement points raises the energy barrier for intermolecular sliding and separation, thereby enhancing the bulk resistance to failure.

The works of adhesion between the sealants and aggregates are shown in Figure 5d. With respect to aggregate type, the work of adhesion exhibits a pronounced mineral-type dependence. Values for the basalt system ranged from 39.33 to 79.18 mJ/m^2^, whereas those for the granite system ranged from only 16.48 to 42.06 mJ/m^2^, with the former being 1.88–2.43 times the latter. Basalt presents an alkaline, chemically active surface that wets readily with the sealants, giving the smallest sealant–aggregate contact angles and correspondingly the highest work of adhesion. Granite, an acidic aggregate with a high SiO_2_ content, wets less favorably and yields lower work of adhesion at the interface. Notably, basalt exhibits the lowest total surface free energy among the three aggregates yet yields the highest work of adhesion, indicating that sealant–aggregate adhesion is governed primarily by the chemical character of the aggregate surface rather than by its total surface free energy.

With respect to material type, sealant C achieved the highest work of adhesion with all three aggregates, indicating that its interfacial adhesion capacity is independent of aggregate type and that it exhibits broad mineral adaptability.

#### 3.1.3. Pull-Off Strength

**Figure 6:**
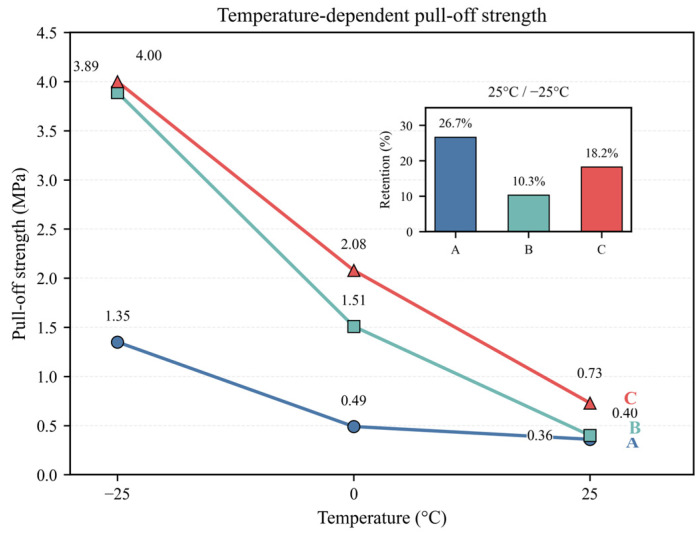
Pull-off strength at different temperatures with strength retention inset.

The pull-off strengths of the three crack sealants at −25 °C, 0 °C, and 25 °C are shown in Figure 6. As temperature increased from −25 °C to 25 °C, the pull-off strength of sealant A decreased from 1.35 to 0.36 MPa, that of sealant B from 3.89 to 0.40 MPa, and that of sealant C from 4.00 to 0.73 MPa, corresponding to reductions of 73%, 89%, and 82%, respectively. Rising temperature intensifies molecular chain segment motion and expands the free volume of the sealant, simultaneously weakening van der Waals forces and polar interactions at the interface, which leads to a reduction in bond strength. The pull-off strength ranking remained C > B > A at all test temperatures, consistent with the ranking of the work of adhesion, confirming that the work of adhesion calculated from contact angle measurements and surface free energy theory is an effective predictor of actual interfacial bond strength. The pull-off strength of sealant C at 25 °C was still 2.03 times that of sealant A, demonstrating that the higher modifier content maintains effective interfacial load-bearing capacity even at elevated temperatures. Taking the strength at −25 °C as the reference, the strength retention ratios of sealants A, B, and C at 25 °C were 26.7%, 10.3%, and 18.2%, respectively; sealant B exhibited the lowest retention ratio, indicating the greatest sensitivity to temperature increase. However, the retention ratio should be interpreted in conjunction with absolute strength values: sealant C achieved the highest absolute pull-off strength at all test temperatures, demonstrating the best overall resistance to temperature-induced degradation. Diurnal temperature swings at high-altitude airports can reach 30–40 °C, requiring the sealant to maintain effective interfacial bonding over the full −25 °C to 25 °C range. Sealant C achieved the highest absolute pull-off strength across this entire range, meeting the interfacial load-bearing demands imposed by the extreme thermal cycling of high-altitude airports.

#### 3.1.4. Shear Strength and Failure Work

**Figure 7:**
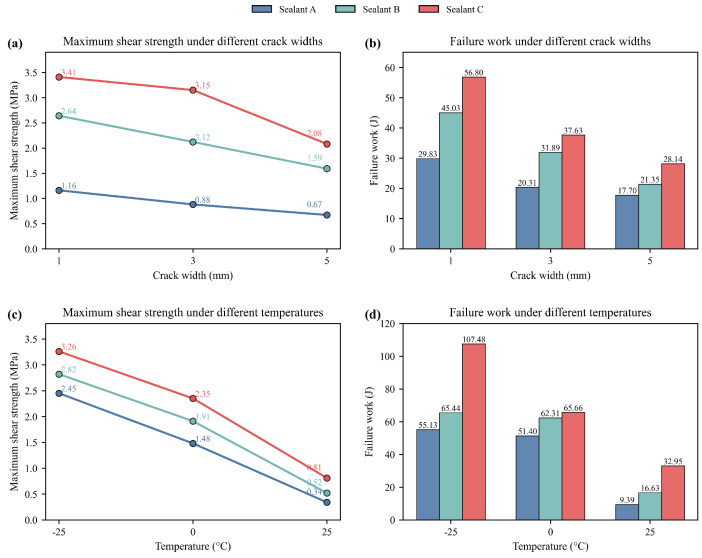
Shear failure resistance of crack sealants: (**a**) maximum shear strength at different crack widths, 0 °C; (**b**) failure work at different crack widths, 0 °C; (**c**) maximum shear strength at different temperatures, 5 mm crack width; (**d**) failure work at different temperatures, 5 mm crack width.

The maximum shear strengths and failure works of the three crack sealants at crack widths of 1, 3, and 5 mm under isothermal conditions at 0 °C are shown in Figure 7a,b. As crack width increased from 1 to 5 mm, the maximum shear strength decreased by 42.35%, 44.20%, and 45.72% for sealants A, B, and C, respectively, while failure work decreased by 40.66%, 52.59%, and 50.46% for sealants A, B, and C, respectively. Increasing crack width reduces the effective contact area between the sealant and the crack walls, lowering interfacial frictional resistance; concurrently, a wider sealant layer is more susceptible to stress concentration under shear loading, making the critical conditions for crack initiation easier to satisfy. These two effects together lead to a systematic deterioration in shear load-bearing capacity. Across the range of crack widths tested, the mean shear strength of sealant C was 46.46% and 26.88% higher than that of sealants A and B, respectively, while the mean failure work of sealants A and B was 44.65% and 19.83% lower than that of sealant C. This advantage is attributed to the thermoplastic elastomer network formed by the higher SBS content in sealant C, which absorbs greater elastic strain energy through reversible extension of molecular chain segments during shear deformation, thereby retarding crack propagation to critical failure. Cracks in high-altitude airport pavements propagate continuously under thermal contraction and temperature cycling, with crack widths expanding from an initial 1 mm to 5 mm or beyond during service. Sealant C maintained the highest shear strength and failure work across the full range of crack widths tested, satisfying the mechanical demands throughout the entire crack development process.

The maximum shear strengths and failure works of the three crack sealants at −25 °C, 0 °C, and 25 °C under a 5 mm crack width are shown in Figure 7c,d. As temperature increased from −25 °C to 25 °C, shear strength decreased by 86.12%, 81.56%, and 71.88% for sealants A, B, and C, respectively. At low temperatures, the free volume of the sealant contracts and molecular chain segment mobility is restricted; the material exhibits glassy, brittle behavior with failure dominated by sudden interfacial delamination, corresponding to high peak loads and low critical displacements. As temperature rises, increased chain segment mobility drives a transition from brittle to viscoelastic behavior; the decay in interfacial viscosity weakens the frictional interlocking effect, and the failure mode shifts to progressive viscous flow. The shear strength reduction of sealant C was 71.88%, lower than the 86.12% of sealant A and the 81.56% of sealant B; furthermore, the failure work of sealant C at 25 °C remained 3.51 times that of sealant A. These results indicate that the higher SBS and crumb rubber contents, through the formation of a dense physical cross-linking network, effectively suppressed viscous flow of molecular chains at elevated temperatures, conferring superior shear failure resistance across a wide temperature range.

### 3.2. Microstructural Characteristics of Crack Sealants

#### 3.2.1. Fluorescence Microscopy Phase Morphology

**Figure 8:**
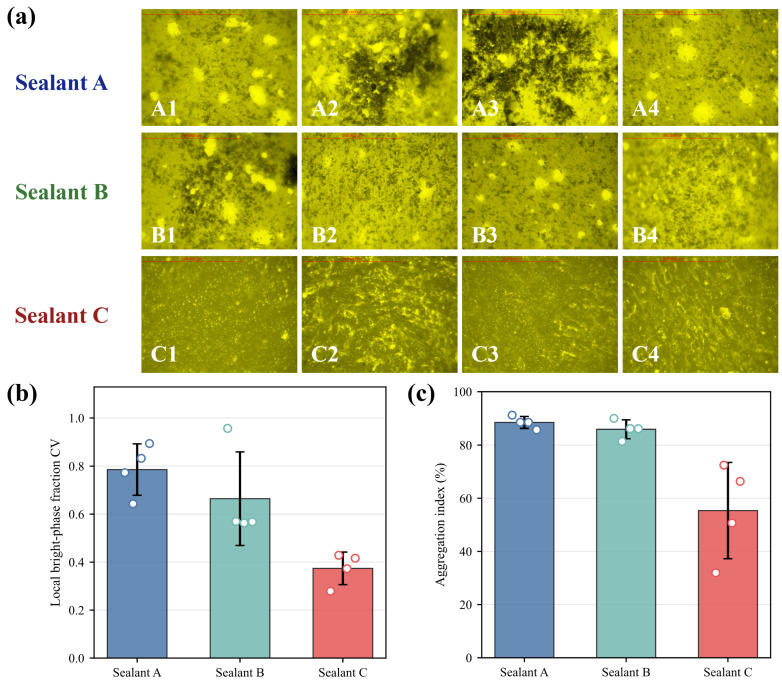
Fluorescence microstructure and quantitative dispersion characteristics of crack sealants: (**a**) fluorescence micrographs of four replicate fields of view for sealants A (A1–A4), B (B1–B4), and C (C1–C4); (**b**) local bright-phase fraction CV; (**c**) aggregation index.

The fluorescence microscopy images and quantitative dispersion characteristics of the three crack sealants are shown in Figure 8. Sealant A exhibits pronounced local fluorescence-rich regions and dark particle-rich regions, indicating significant microstructural heterogeneity. The dispersion uniformity of sealant B is improved relative to sealant A, but localized aggregation remains. Sealant C shows the most uniform and continuous distribution of the fluorescent phase, with no discernible large-scale agglomerates.

The quantitative image analysis results further corroborate these observations. The coefficients of variation of the local bright-phase fraction CV_φ_ for sealants A, B, and C were 0.786 ± 0.107, 0.664 ± 0.195, and 0.374 ± 0.068, respectively, and the aggregation indices AI were 88.5% ± 2.2%, 85.9% ± 3.5%, and 55.4% ± 18.1%, respectively. The CV_φ_ of sealant C was 52.4% lower than that of sealant A, and AI was 37.4% lower, quantitatively confirming that modifier dispersion uniformity in the asphalt matrix is markedly superior to that of sealants A and B. Although sealant C contains the highest SBS and crumb rubber contents, the fluorescent phase still presents a fine, uniform distribution rather than large-scale agglomeration, indicating that good compatibility between the modifier and the asphalt matrix is maintained at higher dosage levels. This uniform and continuous microstructural phase morphology increases the interfacial area between the polymer phase and the asphalt matrix and enhances molecular chain entanglement density, constituting the microstructural basis for the highest work of cohesion and interfacial adhesion strength exhibited by sealant C. A sensitivity analysis across four percentile thresholds (top 10–25%) preserved the rank order for both descriptors, and a Kruskal–Wallis test confirmed significant intersealant differences (CV_φ_: *p* = 0.018; AI: *p* = 0.021).

#### 3.2.2. FTIR Chemical Composition Analysis

**Figure 9:**
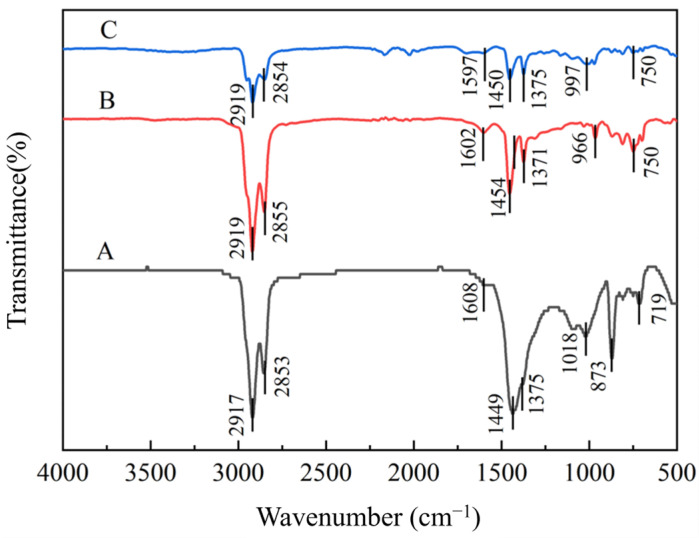
FTIR spectra of crack sealants.

The FTIR spectra of the three crack sealants are shown in Figure 9. All three exhibit strong absorption bands at 2920 cm^−1^ and 2853 cm^−1^ assigned to saturated C-H stretching vibrations, confirming the presence of alkyl chains or saturated hydrocarbon components. The characteristic band near 1600 cm^−1^ is assigned to aromatic C=C skeletal stretching vibrations, indicating the presence of aromatic ring structures. The bands at 1450 cm^−1^ and 1375 cm^−1^ are attributed to the asymmetric and symmetric bending vibrations of methyl -CH_3_ groups; all three sealants thus exhibit a chemical structure characterized by the coexistence of aromatic and aliphatic components. A characteristic band at 966 cm^−1^, assigned to the trans C=C-H out-of-plane bending vibration of the butadiene segments in SBS, confirms that the SBS modifier has been successfully incorporated into the asphalt matrix. This band is progressively more pronounced from sealant A to C, consistent with their increasing SBS content. The positions and types of characteristic bands are consistent across the three sealants, indicating that they share the same principal functional-group types. Taken together with the fluorescence microscopy results, the differences in macroscopic performance among the three sealants do not originate from fundamental differences in chemical composition but are governed by modifier content and its microscale dispersion state in the asphalt matrix. Higher modifier content combined with more uniform dispersion produces a denser intermolecular physical cross-linking network, which in turn enhances both cohesive strength and interfacial adhesion capacity.

### 3.3. MD Simulation of Adhesion Performance

#### 3.3.1. Validation of Molecular Models

**Figure 10:**
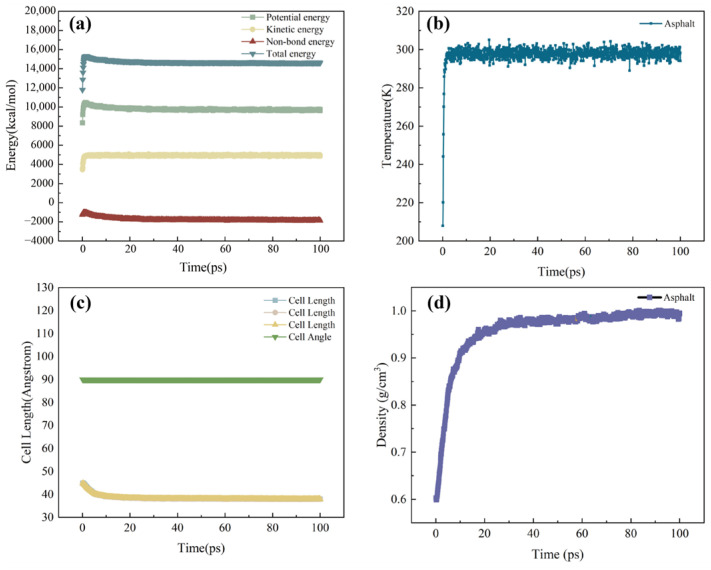
Convergence profiles during NPT equilibration of the asphalt model: (**a**) potential energy, (**b**) temperature, (**c**) cell edge length and cell angles, (**d**) density.

**Figure 11:**
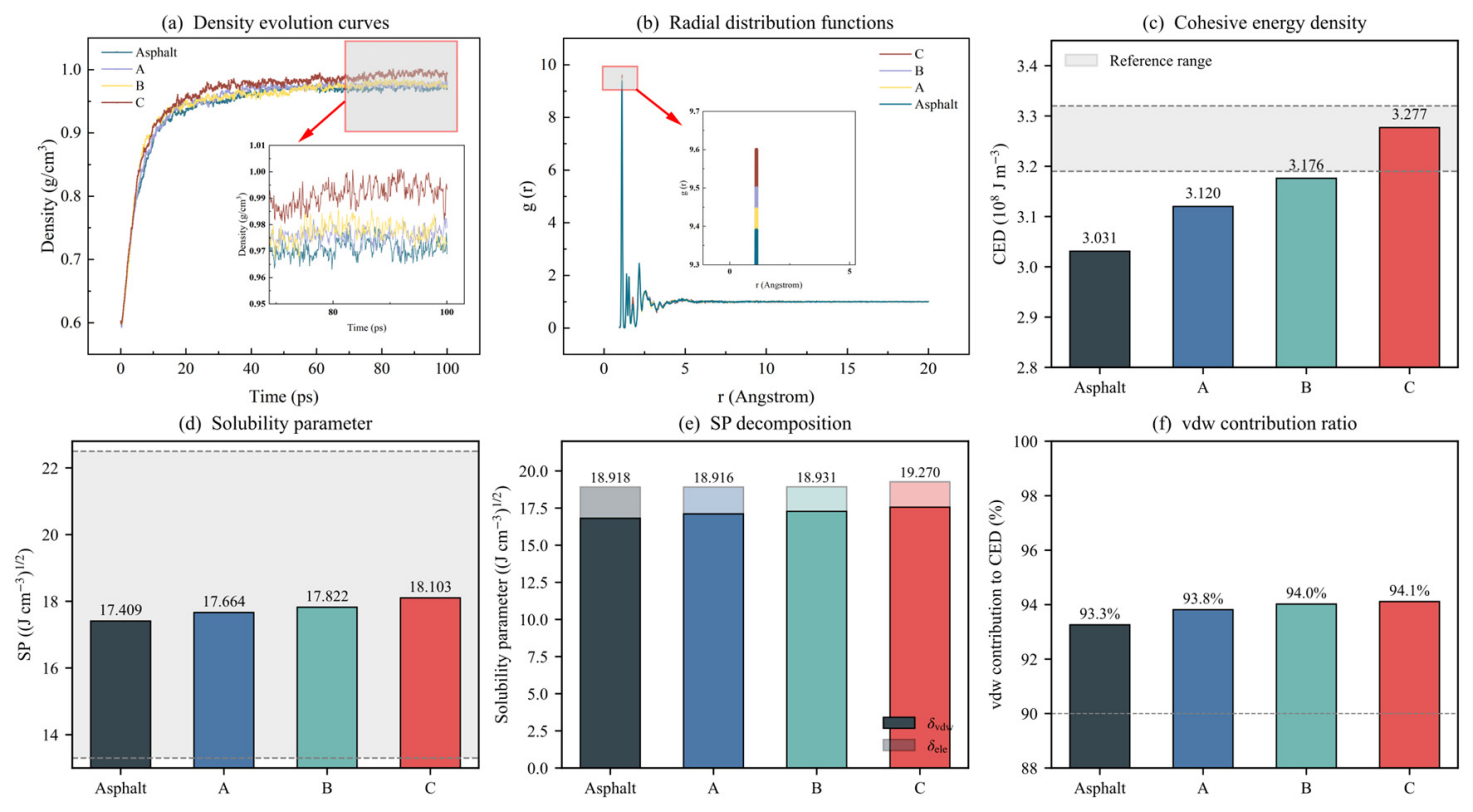
Molecular model validation: (**a**) density, (**b**) RDF, (**c**) CED, (**d**) solubility parameter, (**e**) SP decomposition, (**f**) van der Waals contribution ratio. The gray shaded areas denote reference ranges for asphalt binders.

The convergence of the system during equilibration is shown in Figure 10. Taking the asphalt model as a representative example, the potential energy, box edge length, and density all reached stable plateaus concurrently, while the cell angles remained at 90° and the temperature stabilized at 298 K (fluctuation < 5 K); the box contracted from an initial state (density ≈ 0.60 g/cm^3^) to an equilibrium cubic cell of 37.98 Å per edge, confirming that dynamic equilibrium had been attained. The three sealant models followed an identical protocol. The density evolution curves following NPT ensemble equilibration are shown in Figure 11a, and the equilibrium densities are listed in Table 9. The asphalt model density converged to 0.9698 g/cm^3^, which falls within the reference range of 0.9500–1.0800 g/cm^3^ for real asphalt at this temperature, with a deviation of less than 2%. The equilibrium densities of the three sealant models were 0.9704, 0.9793, and 0.9937 g/cm^3^, respectively, increasing with SBS and crumb rubber content; this trend is consistent with the physical process whereby modifiers fill intermolecular voids and reduce free volume in actual crack sealants. The simulated sealant densities are approximately 3–7% below the reported range of 1.00–1.05 g/cm^3^ for polymer-modified asphalt [44,48,49], consistent with the typical accuracy of all-atom force-field predictions, where MD-simulated densities are systematically 7–10% lower than experimental values due to force-field approximations and finite non-bonded cutoff effects [48,49]. The remaining discrepancy is further attributable to the exclusion of trace metallic impurities and inorganic fillers from the all-atom models.

The radial distribution functions are shown in Figure 11b. The RDF curves of all three sealants and asphalt display highly consistent trends. Multiple peaks appear in the 0–2 Å range, corresponding to the short-range ordered arrangement of atoms within covalent bond lengths; g(r) progressively decays with diminishing oscillation in the 2–4 Å range, reflecting decreasing structural order beyond the first coordination shell; beyond 4 Å, g(r) converges to unity, indicating the absence of periodic long-range structure and confirming typical amorphous disordered character. This structural feature is fully consistent with the intrinsic properties of asphalt and polymer-modified asphalt as amorphous macromolecular materials. The combined validation across all four parameters confirms that the constructed molecular models are reliable at the levels of density, energy, and structure and are suitable for the subsequent quantitative simulation of interfacial interactions.

The cohesive energy densities and solubility parameters are shown in Figure 11c,d; both CED and solubility parameter follow the increasing trend C > B > A across the three sealants. As shown in Figure 11e,f, the van der Waals component δ_vdw_ is substantially greater than the electrostatic component δ_ele_, and the square of the van der Waals component accounts for more than 90% of the total CED, indicating that cohesion in the asphalt and crack sealant systems is dominated by non-polar dispersion interactions. Sealant C exhibits the highest CED, which is consistent with stronger overall cohesive interactions and provides an energetic explanation for its highest work of cohesion. Furthermore, the solubility parameter differences between each sealant and the asphalt matrix are all less than 2.0 (J/cm^3^)^0.5^, indicating good thermodynamic compatibility among the three sealants and the asphalt matrix and no risk of macroscopic phase separation due to insufficient compatibility.

#### 3.3.2. Mean Square Displacement and Diffusion Coefficients

**Figure 12:**
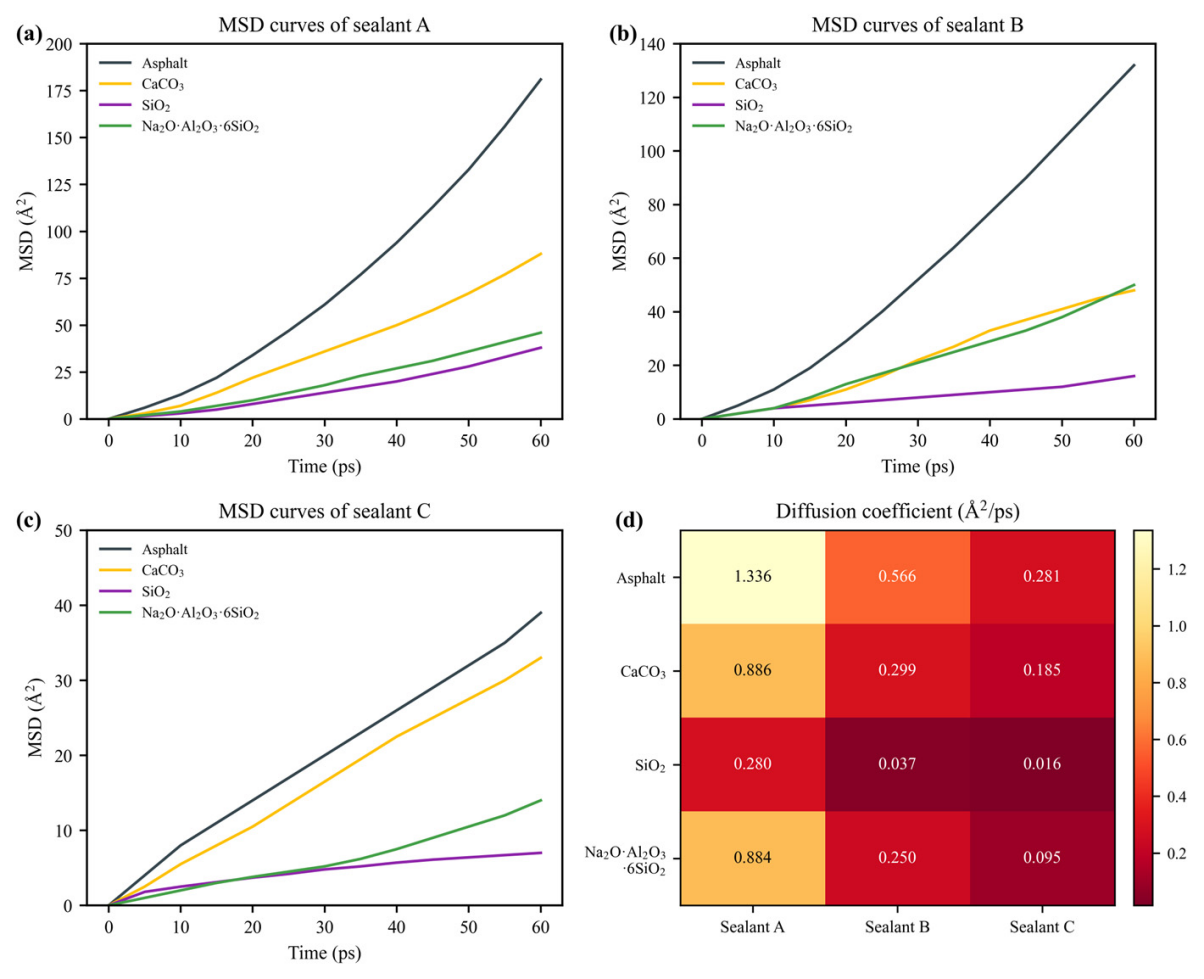
Molecular migration and diffusion behavior: (**a**) MSD curves of sealant A; (**b**) MSD curves of sealant B; (**c**) MSD curves of sealant C; (**d**) diffusion coefficients.

The MSD curves of the three crack sealant molecules at aggregate surfaces over 0–60 ps at 298 K are shown in Figure 12a–c, and the corresponding diffusion coefficients are presented in Figure 12d. Within the simulation time window, MSD increased monotonically with time at a nearly constant slope, indicating that the molecules were in the normal diffusion regime and satisfied the linear assumption of the Einstein relation. The MSD values followed the order A > B > C, as did the corresponding diffusion coefficients. Taking the SiO2 interface as an example, the diffusion coefficient of sealant A was 0.280 Å^2^/ps, whereas that of sealant C was only 0.016 Å^2^/ps, the diffusion coefficient of sealant A being 17.5 times that of sealant C.

From a molecular structural perspective, the differences in MSD directly reflect the varying degrees of freedom of molecular chain segment motion among the three sealants. Sealant A contains the highest asphalt content of 80%; its molecular chain segments are relatively short and of low molecular weight, with sparse intermolecular entanglement points, allowing segments to move freely over a large range and thus yielding the highest MSD. In sealant C, the SBS content reaches 15% and the crumb rubber content 20%: the styrene blocks of SBS form physical cross-linking nodes through microphase separation, the butadiene blocks constitute elastic connecting chains between these nodes, and crumb rubber particles further fill intermolecular voids and restrict chain segment migration pathways. These three components cooperatively build a dense constraint network that significantly suppresses molecular chain segment motion, resulting in the lowest MSD. A smaller MSD corresponds to more restricted chain segment mobility and a longer residence time of sealant molecules near the aggregate surface, which is favorable for the development of stable interfacial adsorption layers. Taken together with the interface-energy and macroscopic strength results, this restricted mobility is consistent with the stronger interfacial bonding observed for sealant C.

With respect to aggregate type, the diffusion coefficient of a given sealant at different aggregate surfaces follows the order asphalt > CaCO_3_ > Na_2_O·Al_2_O_3_·6SiO_2_ > SiO_2_. Taking sealant A as an example, its diffusion coefficient at the asphalt surface was 1.336 Å^2^/ps, whereas at the SiO_2_ surface it was only 0.280 Å^2^/ps, a reduction of 79.0%. The SiO_2_ surface contains abundant Si-O dangling bonds that form strong surface adsorption interactions with the polar groups of sealant molecules, imposing the greatest constraint on molecular motion and yielding the lowest diffusion coefficient. The presence of alkali metal ions in Na_2_O·Al_2_O_3_·6SiO_2_ further intensifies the surface electrostatic field, exerting directional attraction on the polar components of the sealant; its diffusion coefficient is therefore close to that of SiO_2_. The surface polarity of CaCO_3_ is comparatively weaker, resulting in moderate constraints on molecular motion. These results indicate that higher surface chemical activity of the aggregate leads to greater restriction of sealant molecular migration in the interfacial region and tighter interfacial bonding. The above differences in diffusion behavior provide a molecular-level explanation for the experimentally observed higher work of adhesion of alkaline aggregates compared with acidic aggregates in Section 3.1.2. Basalt and other alkaline aggregates are commonly selected for high-altitude airport pavements; the MD results demonstrate that such aggregates impose the strongest constraint on sealant molecular migration, confirming the suitability of alkaline aggregates for crack sealing applications from the molecular perspective.

#### 3.3.3. Surface Free Energy and Work of Cohesion

**Figure 13:**
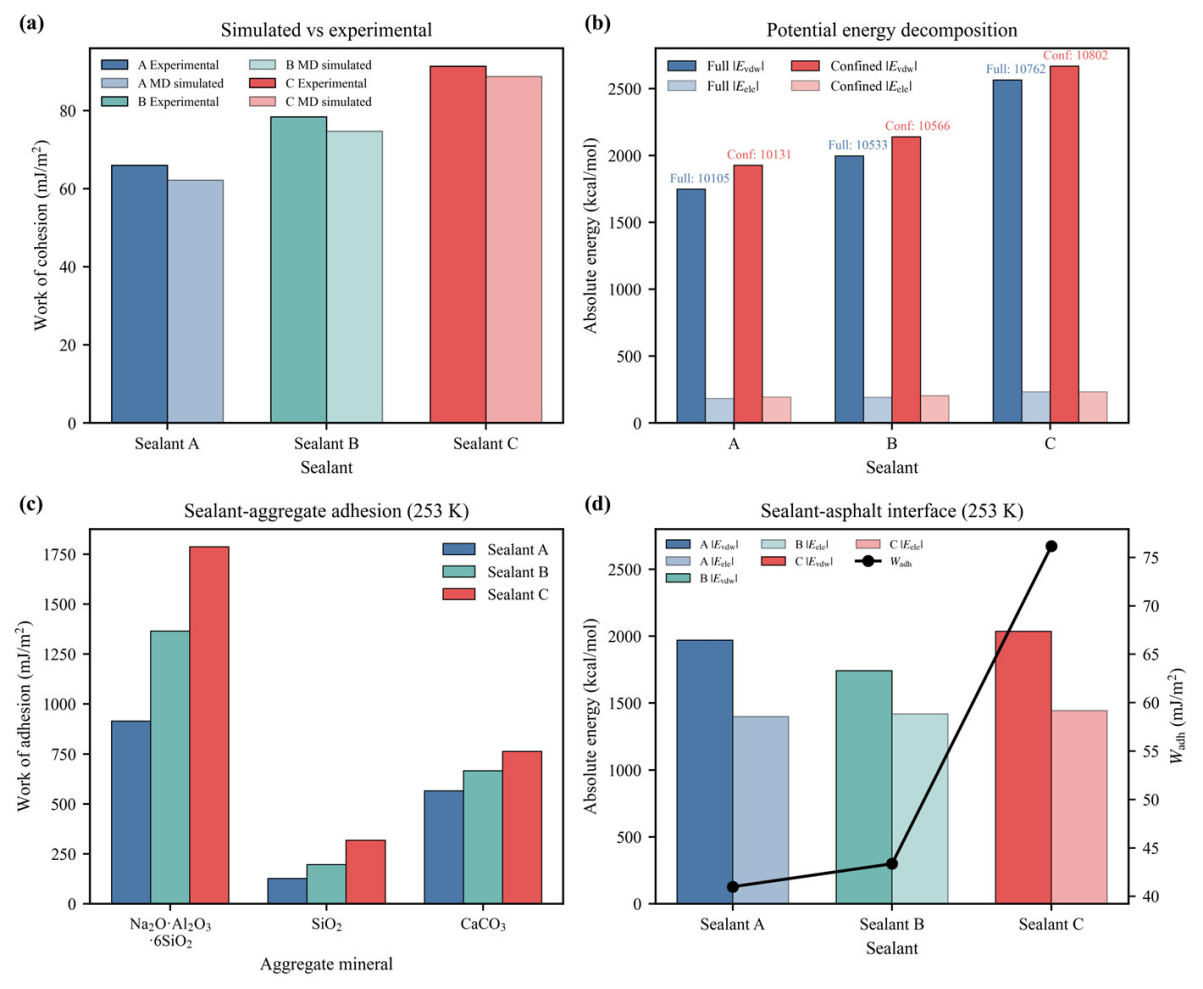
Adhesion energy analysis: (**a**) simulated vs. experimental cohesion work; (**b**) potential energy decomposition of sealant models; (**c**) sealant–aggregate interface energy and adhesion work; (**d**) sealant–asphalt interface energy decomposition and adhesion work.

The surface free energy and work of cohesion of the three crack sealants were calculated from the potential energy difference between the full-periodic and confined models using Equation (13) and are presented in Figure 13a; the potential energy decomposition of each model is shown in Figure 13b. The total potential energies of both the full-periodic and confined models followed the order C > B > A. Analysis of the potential energy components showed that van der Waals interactions consistently dominated the total potential energy, with electrostatic contributions comparatively small, consistent with the CED analysis in Section 3.3.1 and further confirming that intermolecular cohesion in crack sealant systems is governed primarily by dispersion forces. The absolute van der Waals potential energy of the full-periodic model of sealant C was 2563.18 kcal/mol, 46.7% higher than that of sealant A (1747.12 kcal/mol), indicating that the higher SBS and crumb rubber contents substantially strengthened intermolecular non-bonded interactions.

The simulated work of cohesion followed the order C > B > A, with sealant C yielding 88.77 mJ/m^2^, 42.7% higher than sealant A (62.21 mJ/m^2^). Comparison with the experimental values from Section 3.1.2 revealed identical ranking; the relative deviation between simulated and experimental values was 2.88–5.74%. The systematic underestimation is attributed to the simplified treatment of saturates and asphaltenes in the model construction, which causes partial underestimation of non-bonded intermolecular interactions. Nevertheless, the simulated values were numerically close to the experimental results and preserved the relative differences and ranking among the three sealants. This agreement between experiment and simulation confirms that molecular dynamics simulation serves as a reliable surrogate for evaluating the cohesive properties of crack sealants and provides a theoretical tool for rapid performance screening in the material development stage.

#### 3.3.4. Sealant–Aggregate Interface Energy and Work of Adhesion

The trends in interface energy and work of adhesion between the three crack sealants and three aggregate surfaces are presented in Figure 13c. Potential energy decomposition showed that, although both van der Waals and electrostatic interactions contributed to the interfacial interaction, the electrostatic contribution was dominant and negative, indicating that electrostatic attraction is the primary driving force for sealant–aggregate adhesion. Within the sealant bulk, by comparison, cohesion is governed mainly by van der Waals interactions (Section 3.3.3). Across aggregate types, the work of adhesion with Na_2_O·Al_2_O_3_·6SiO_2_ was far higher than with SiO_2_ or CaCO_3_. For sealant C at 293 K, the values were 1678.11, 756.15, and 217.58 mJ/m^2^ for Na_2_O·Al_2_O_3_·6SiO_2_, CaCO_3_, and SiO_2_, respectively, the first being 7.71 times the last. The metal cations exposed on the aluminosilicate surface intensify the surface electrostatic field and produce strong Coulombic attraction with the polar components of the sealant, giving the highest interfacial binding energy among the three minerals. This electrostatic field is carried by Na^+^ in the feldspar model and by Ca and Mg in the plagioclase and pyroxene phases of natural basalt, so the alkaline aluminosilicate surface provides the electrostatically active sites associated with interfacial adhesion. This shift in the dominant interfacial interaction with mineral type, from dispersion-controlled bonding on quartz-type surfaces to electrostatically controlled bonding on feldspar-type surfaces, is consistent with previous molecular simulations of asphalt–aggregate interfaces [45]. This result is consistent with the experimental work of adhesion trend in Section 3.1.2, further confirming the interfacial adhesion advantage of alkaline aggregates from the perspective of interface energy.

Interface energy and work of adhesion generally decreased with increasing temperature, though temperature sensitivity varied by aggregate: the SiO_2_ interface was the most thermally stable, the CaCO_3_ interface showed a local maximum at 273 K, and the Na_2_O·Al_2_O_3_·6SiO_2_ interface had the largest absolute work of adhesion but the highest temperature sensitivity. Across all temperatures, sealant C consistently yielded the highest work of adhesion with every aggregate. At the Na_2_O·Al_2_O_3_·6SiO_2_ interface at 253 K, its work of adhesion was 1786.56 mJ/m^2^, 95.3% higher than that of sealant A (914.60 mJ/m^2^) and 30.9% higher than that of sealant B (1365.02 mJ/m^2^). The higher SBS and crumb rubber contents in sealant C not only enhanced bulk cohesive strength but also strengthened electrostatic interaction with aggregate surfaces by raising the polar component content, achieving synergistic enhancement of both cohesion and interfacial adhesion. The simulation temperature of 253 K corresponds to extreme winter low-temperature conditions at high-altitude airports. Under this condition, sealant C still exhibited the highest work of adhesion, indicating that it can maintain strong interfacial bonding under extreme low temperatures and is less susceptible to low-temperature debonding failure.

#### 3.3.5. Sealant–Asphalt Interface Energy and Work of Adhesion

The interface energy and work of adhesion of the three sealant–asphalt interface models at 253 K are presented in Figure 13d. The interface energy of sealant C was 185.61 kcal/mol, 91.4% and 85.5% higher than that of sealant A (96.97 kcal/mol) and sealant B (100.05 kcal/mol), respectively. The corresponding work of adhesion was 76.15 mJ/m^2^, 85.8% and 75.7% higher than that of sealants A and B, respectively.

Analysis of the potential energy components showed that both van der Waals and electrostatic interactions contributed to the work of adhesion at the sealant–asphalt interface; unlike the sealant–aggregate interface where electrostatic forces were dominant, however, the van der Waals contribution at the sealant–asphalt interface slightly exceeded the electrostatic contribution. This difference arises because asphalt is an organic system in which non-polar interactions predominate, so dispersion forces govern sealant–asphalt interaction, whereas the ionic mineral structure of aggregate surfaces makes electrostatic attraction the primary driving force at the sealant–aggregate interface. The substantially higher work of adhesion of sealant C at the sealant–asphalt interface indicates that sealant C not only forms strong bonds with bare aggregate but also maintains effective adhesion with existing asphalt layers on crack walls. In practical pavement crack repair, a crack sealant must simultaneously bond to both aggregate and aged asphalt surfaces; sealant C exhibited superior adhesion at both interfaces, which can effectively reduce the risk of interfacial debonding-induced adhesion failure during service.

### 3.4. Multi-Scale Correlation Analysis

For each indicator measured at both scales, the rankings of the three sealants from surface free energy measurements and from MD simulation were compared. Across surface free energy, work of cohesion, and work of adhesion on granite, limestone, and basalt, both scales ranked the formulations in the same order (C > B > A), with the works of cohesion also agreeing in magnitude to within 6%. The absolute works of adhesion from MD were markedly higher than the experimental values, as the molecular models adopt idealized crystal surfaces and neglect the roughness, impurities, and weathering layers of real aggregates, yet the ranking remained consistent across scales. The molecular-scale energy parameters showed the same order (C > B > A) as the pull-off and shear strengths, indicating that the molecular-scale ranking is preserved at the macroscopic scale. As only three formulations were compared (n = 3), these agreements are treated descriptively rather than inferentially. The three formulations are ranked in exactly the same order at both scales (complete ordinal agreement, Spearman *ρ* = 1.0), while the linear closeness of the paired values varies with the indicator (Pearson r ranging from about 0.89 to 1.00). They are therefore interpreted as corroborative evidence that MD simulation can support the relative comparison and screening of interfacial adhesion, rather than as a validated predictive correlation.

These results indicate a consistent cross-scale ranking of the three formulations, suggesting that MD simulation can serve as a useful auxiliary tool for the rapid comparative evaluation of the interfacial energy parameters of crack sealants. Given the small sample (n = 3), this cross-scale consistency is regarded as descriptive corroborative evidence rather than statistical proof of a cross-scale adhesion mechanism. Establishing a predictive cross-scale correlation would require a larger and more diverse set of sealant formulations.

### 3.5. Multi-Scale Analysis of Adhesion Performance

Integrating the findings from each scale, interfacial adhesion failure in crack sealants can be described as a multi-scale coupled process propagating progressively from microstructural phase morphology to macroscopic mechanical response. At the microscale, fluorescence microscopy confirmed that SBS phase particle size distribution and filler dispersion uniformity are the fundamental determinants of structural integrity. As shown in Section 3.2.1, the CV_φ_ and AI of sealant C were 0.374 and 55.4%, 52.4% and 37.4% lower than those of sealant A, confirming markedly superior phase dispersion uniformity. Regions of non-uniform dispersion serve as stress concentration sites and crack initiation locations. FTIR confirmed that the three sealants share the same principal functional-group types, with performance differences governed mainly by modifier content and microscale dispersion state. The failure threshold is therefore set during preparation, and temperature and loading during service are merely external triggers. At the molecular scale, microstructural differences influence interfacial behavior through two pathways. Along the cohesion pathway, the CED of sealant C was 3.277 × 108 J/m^3^, 5.0% above that of sealant A, and its MD work of cohesion was 88.77 mJ/m^2^, 42.7% above that of sealant A, substantially raising the energy barrier to bulk fracture. Along the adhesion pathway, the diffusion coefficient of sealant C at the SiO_2_ surface was only 0.016 Å^2^/ps, 94.3% lower than that of sealant A; interface energy analysis further revealed that sealant–aggregate adhesion is dominated by electrostatic forces while sealant–asphalt adhesion is dominated by van der Waals forces, yet sealant C exhibited the highest binding energy at both interfaces. The synergistic enhancement along both pathways means that sealant C requires the highest critical energy for either cohesive failure or interfacial debonding.

At the macroscale, pull-off and shear strength both followed the order C > B > A; the experimental and molecular-scale indicators followed a consistent ordering across scales, which is consistent with a level-by-level transmission from microstructure to macroscopic response. Under identical degradation conditions, the temperature-induced reduction in shear strength of sealant C was 71.88%, lower than 86.12% for sealant A and 81.56% for sealant B, indicating that the dense physical cross-linking network of sealant C effectively retards the deterioration of interfacial adhesion under both thermal and geometric loading. In summary, microstructural phase dispersion uniformity, intermolecular interaction strength, interfacial energy parameters, and macroscopic mechanical load-bearing capacity increase in concert in a progressive, level-by-level manner. Increasing modifier content while ensuring uniform dispersion is closely associated with synchronized improvement across all hierarchical levels, providing a directional reference for optimizing crack sealant formulations.

### 3.6. CRITIC-TOPSIS Comprehensive Evaluation

**Figure 14:**
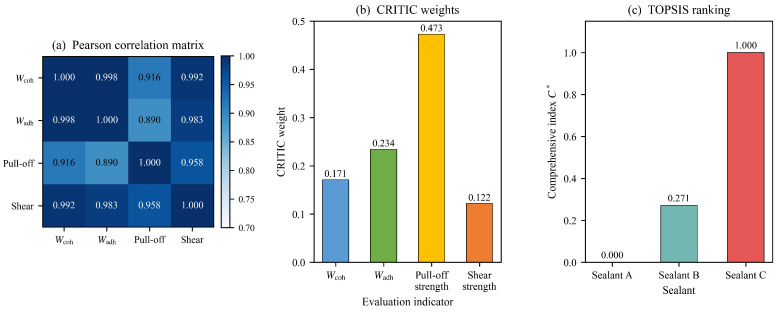
CRITIC-TOPSIS evaluation: (**a**) Pearson correlation matrix, (**b**) CRITIC objective weights, (**c**) comprehensive performance index C*.

The three crack sealants were evaluated using CRITIC-TOPSIS based on four indicators measured at 25 °C, namely work of cohesion, mean work of adhesion, pull-off strength, and shear strength. The Pearson correlation matrix and the CRITIC weights after min–max normalization are shown in Figure 14a,b. All four indicators increased consistently with modifier content and were highly consistent across formulations (Pearson r = 0.890 to 0.998). The CRITIC method assigned weights of 0.473 to pull-off strength, 0.234 to work of adhesion, 0.171 to work of cohesion, and 0.122 to shear strength. The two indicators that directly characterize interfacial bonding, pull-off strength and work of adhesion, together received about 71% of the total weight, so the objective weighting placed the primary emphasis on interfacial adhesion, in agreement with the multi-scale failure mechanism in which interfacial debonding governs the service performance of crack sealants.

The TOPSIS ranking results are presented in Figure 14c. Sealant C ranked highest on all four indicators and coincided with the positive ideal solution, while sealant A ranked lowest on all four and coincided with the negative ideal solution, giving comprehensive performance indices C* of 1.000 and 0.000 respectively, with sealant B at 0.271 in between. The ranking of C > B > A is consistent with the cross-scale failure mechanism analysis and indicates that sealant C is the preferred candidate material for crack repair of high-altitude airport asphalt pavements within the scope of this study.

## 4. Conclusions

Three low-temperature composite-modified asphalt crack sealants were characterized through surface free energy measurements, mechanical testing, microstructural analysis, and MD simulation. The main conclusions are as follows.

1. Work of cohesion and adhesion increased monotonically with modifier content; the work of cohesion of sealant C exceeded that of sealant A by 38.5%, and alkaline aggregate interfaces yielded 1.88–2.43 times the adhesion of acidic interfaces. Pull-off and shear strength ranked C > B > A, consistent with the adhesion hierarchy.
2. Both temperature rise and crack widening degraded interfacial performance. The shear strength reduction of sealant C across the tested temperature range was 71.88%, substantially lower than 86.12% and 81.56% for sealants A and B, reflecting effective chain segment constraint by its denser cross-linking network.
3. FTIR confirmed that the three sealants share the same principal functional-group types; performance differences originate from modifier content and its dispersion state. The CV_φ_ and AI of sealant C were 52.4% and 37.4% lower than those of sealant A, establishing uniform phase distribution as the microstructural basis of its performance advantage.
4. Simulated works of cohesion deviated from experimental values by only 2.88–5.74%. Sealant–aggregate adhesion is electrostatically dominated; sealant–asphalt adhesion is van der Waals dominated. The experimental and simulated indicators showed a consistent ordering from microstructural morphology to macroscopic mechanical response.
5. CRITIC-TOPSIS yielded C* of 1.000, 0.271, and 0.000 for sealants C, B, and A, respectively, identifying sealant C as the optimal material for crack repair of high-altitude airport asphalt pavements.

## Figures and Tables

**Table 1:** Mass fraction of main components of crack sealants.

Sealant	SBS/%	Crumb Rubber/%	Asphalt/%
A	5	15	80
B	8	17	75
C	15	20	65

**Table 2:** Basic technical properties of crack sealants.

Property	Unit	Requirement	A	B	C
Cone penetration	0.1 mm	70–110	86.0	103.7	90.0
Softening point	°C	≥80	86.5	85.5	90.0
Flow value	mm	≤5	1.1	3.4	1.0
Elastic recovery	%	30–70	38.0	34.7	60.0
Low-temperature tensile ^a^	N/A	−20 °C, 100%, 3 cycles, pass	Pass	Pass	Pass

^a^ Elongations of 25%, 50%, 100%, 150%, and 200% correspond to displacements of 3.75, 7.5, 15, 22.5, and 30 mm, respectively.

**Table 3:** Surface energy parameters of probe liquids.

Liquid	$\gamma_{l}$/(mJ/m^2^)	$\gamma_{l}^{d}$/(mJ/m^2^)	$\gamma_{l}^{p}$/(mJ/m^2^)
Distilled water	72.80	21.80	51.00
Ethylene glycol	47.50	29.00	18.50
Formamide	58.00	39.00	19.00

**Table 4:** Molecular model parameters of asphalt.

Molecule	SARA Fraction	Molecular Formula	Molar Mass/(g/mol)	Number	Mass Fraction/%
SA-1	Saturates	C_30_H_62_	422	4	5.2
SA-2		C_35_H_62_	482	4	5.8
AR-1	Aromatics	C_35_H_44_	464	11	15.7
AR-2		C_30_H_46_	406	13	16.2
RE-1	Resins	C_36_H_57_N	503	4	6.4
RE-2		C_40_H_60_S	572	4	7
RE-3		C_18_H_10_S_2_	290	15	13.4
RE-4		C_40_H_59_N	553	4	6.8
RE-5		C_29_H_50_O	414	5	6.2
AS-1	Asphaltenes	C_42_H_54_O	574	3	5.3
AS-2		C_66_H_81_N	887	2	5.5
AS-3		C_51_H_62_S	706	3	6.5

**Table 5:** Composition of SBR model.

Component	Molecular Formula	Number of Repeat Units	Mass Fraction/%
Styrene	C_8_H_8_	4	39.1
Butadiene	C_4_H_6_	12	60.9

**Table 6:** Lattice parameters of aggregate unit cells.

Aggregate Type	Lattice Parameters					
	Length Parameters/Å			Angle Parameters/°		
	*a*	*b*	*c*	*α*	*β*	*γ*
Na_2_O·Al_2_O_3_·6SiO_2_	7.17	7.42	8.03	110.6	115.89	100.48
SiO_2_	4.91	4.91	5.43	90	90	120
CaCO_3_	5.01	5.01	16.99	90	90	120

**Table 7:** Supercell parameters of aggregate models.

Mineral Type	U Multiplier	V Multiplier	Supercell Dimensions/Å		
			*a*	*b*	*c*
Na_2_O·Al_2_O_3_·6SiO_2_	6	6	43.032	43.032	39.201
SiO_2_	9	9	44.235	44.235	37.536
CaCO_3_	9	9	46.125	46.125	40.052

**Table 8:** Contact-angle-derived thermodynamic adhesion parameters of crack sealants.

Parameter	Sealant A	Sealant B	Sealant C
$\gamma_{s}$/mJ·m^−2^	33.00	39.23	45.70
$\gamma_{s}^{d}$/mJ·m^−2^	4.59	5.10	0.65
$\gamma_{s}^{p}$/mJ·m^−2^	28.41	34.13	45.05
$W_{coh}$/mJ·m^−2^	66.00	78.46	91.40
$W_{adhgranite}$/mJ·m^−2^	16.48	27.35	42.06
$W_{adhlimestone}$/mJ·m^−2^	31.36	47.70	66.37
$W_{adhbasalt}$/mJ·m^−2^	39.33	66.57	79.18

**Table 9:** Density parameters of molecular models.

Model	Simulated Density/(g/cm^3^)	Reference Range/(g/cm^3^)
Asphalt	0.9698	0.9500–1.0800 [50]
Sealant A	0.9704	1.00–1.05 [44,48,49]
Sealant B	0.9793	
Sealant C	0.9937	

## Data Availability

The original contributions presented in this study are included in the article. Further inquiries can be directed to the corresponding author.
